# Towards microalga-based superfoods: heterologous expression of zeolin in *Chlamydomonas reinhardtii*

**DOI:** 10.3389/fpls.2023.1184064

**Published:** 2023-05-09

**Authors:** Federico Perozeni, Matteo Pivato, Margherita Angelini, Elisa Maricchiolo, Andrea Pompa, Matteo Ballottari

**Affiliations:** ^1^ Dipartimento di Biotecnologie, Università degli Studi di Verona, Verona, Italy; ^2^ Dipartimento di Scienze Biomolecolari, Università degli Studi di Urbino, Urbino, Italy

**Keywords:** *Chlamydomonas*, protein expression, synthetic biology, microalgae, zein, phaseolin

## Abstract

Microalgae are unicellular photosynthetic organisms that can be grown in artificial systems to capture CO_2_, release oxygen, use nitrogen- and phosphorus-rich wastes, and produce biomass and bioproducts of interest including edible biomass for space exploration. In the present study, we report a metabolic engineering strategy for the green alga *Chlamydomonas reinhardtii* to produce high-value proteins for nutritional purposes. *Chlamydomonas reinhardtii* is a species approved by the U.S. Food and Drug Administration (FDA) for human consumption, and its consumption has been reported to improve gastrointestinal health in both murine models and humans. By utilizing the biotechnological tools available for this green alga, we introduced a synthetic gene encoding a chimeric protein, zeolin, obtained by merging the γ-zein and phaseolin proteins, in the algal genome. Zein and phaseolin are major seed storage proteins of maize (*Zea mays*) and bean (*Phaseolus vulgaris*) that accumulate in the endoplasmic reticulum (ER) and storage vacuoles, respectively. Seed storage proteins have unbalanced amino acid content, and for this reason, need to be complemented with each other in the diet. The chimeric recombinant zeolin protein represents an amino acid storage strategy with a balanced amino acid profile. Zeolin protein was thus efficiently expressed in *Chlamydomonas reinhardtii*; thus, we obtained strains that accumulate this recombinant protein in the endoplasmic reticulum, reaching a concentration up to 5.5 fg cell^-1^, or secrete it in the growth medium, with a titer value up to 82 µg/L, enabling the production of microalga-based super-food.

## 1 Introduction

Food production is one of the key challenges faced in supporting life in extra-terrestrial environments for space exploration. Several constraints are present when planning food production in spacecraft or in enclosed environments under harsh conditions, such as limited water and oxygen on the Moon or Mars, cosmic radiation, requirements for nutrient recovery from wastes, and limited spaces. With increasing population, food demands, and desire for healthier lifestyles, alternative sources of functional foods are important for space exploration and terrestrial applications. In this context, photosynthetic microalgae cultivation could be an interesting opportunity because of their reduced water footprint compared to crops and the possibility of growing in different environmental conditions using wastewater as a nitrogen and phosphorous source. The presence of carbon concentration mechanisms in microalgae and the absence of non-photosynthetic tissue, as in the case of vascular plants, allow microalgae to efficiently assimilate CO_2_, releasing oxygen as a byproduct of the photosynthetic process (Onyeaka et al., 2021). Moreover, the biomass produced by microalgae has considerable nutritional value and contains relatively high concentrations of proteins, polyunsaturated fatty acids, polysaccharides, pigments, vitamins, minerals, phenolic compounds and sterols (Camacho et al., 2019). Microalgae biomass has also been reported to be enriched in copper and iron, potentially matching 21% and 11% of the Required Daily Allowance (RDA) for these metals (Koyande et al., 2019). Therefore, microalgae could be considered to enhance CO_2_ sequestration, recover nutrients from waste, and produce edible biomass, with several species already approved as novel foods by the European Food Safety Association (EFSA) and the U.S. Food and Drug Administration (FDA) (Torres-Tiji et al., 2020; Mendes et al., 2022).

The green alga *Chlamydomonas reinhardtii* is a model organism for microalgae and is one of the most used species for laboratory research purposes (fundamental and applied research) because it has fast growth, does not require expensive supplements or an elaborate system for cultivation, is capable of sexual reproduction, and is easily engineered by genetic manipulation of nuclear, chloroplast, and mitochondrial genomes. The rapid advancements in recombinant protein production in this host suggest that in the near future, it could have an important role in the production of compounds of interest for the pharmaceutical and nutraceutical industries (Masi et al., 2023). In addition, it has recently received the Generally Recognized As Safe (GRAS) certification from the FDA for food applications. Recently, *C. reinhardtii* was reported having a positive effect for gastrointestinal function when used as functional food ingredient in both murine and human models (Fields et al., 2020). Metabolic engineering approaches have been proposed to improve carbon storage in lipids; however, little effort has been made to improve carbon storage in proteins in *C. reinhardtii.*

Seed storage proteins accumulate at high levels in seeds as nitrogen, carbon, and sulfur reserves and are then used during seed germination. They do not have any enzymatic function and accumulate in the protein bodies (Krishnan and Coe, 2001). Seed storage proteins of legumes and cereals are two major sources of proteins for humans. From a nutritional perspective, they complement each other: the storage proteins from legumes are poor in sulfur amino acids, and those from cereals are poor in lysine and tryptophan. Among the different storage proteins, phaseolins and zeins have been extensively studied as the main seed storage proteins in legumes and cereals, respectively (Suárez-Martínez et al., 2016; Khan et al., 2019). Phaseolin, a glycoprotein belonging to the 7S vicilin class, is the major seed storage protein in the common bean. Each phaseolin polypeptide is cotranslationally glycosylated in the endoplasmic reticulum (ER) lumen (De La Fuente et al., 2012). being a trimeric high-mannose glycosylated protein of approximately 150 kDa, containing almost identical monomers with a molecular mass ranging from 45 to 51 kDa and isoelectric points of 5.6 to 5.8 (Sathe, 2016). Maize endosperm zein protein belongs to the prolamin family, which are the most abundant type of proteins stored in cereal seeds such as wheat, maize, sorghum, rice, and barley. Zein are divided into four subfamilies based on their solubility and amino acid composition: α (22 and 19 kDa), β (15-kDa), γ (50 kDa, 27 kDa, and 16 kDa), and δ (18 kDa and 10 kDa) zeins (Holding, 2014), and these proteins are found in protein bodies (PBs) inside the endoplasmic reticulum (ER) (Lending and Larkins, 1989), accounting for more than 60% of the total stored proteins in the endosperm of maize kernels (Llop-Tous et al., 2010; Holding, 2014; Khan et al., 2019). Numerous studies have suggested that the packaging of zein and non-zein proteins into PBs has a peculiar role in maize endosperm development, influencing kernel properties (e.g., texture, functionality, and protein quality) (Guo et al., 2013; Holding, 2014). Due to reciprocal limitations in terms of amino acid composition, phaseolin and γ-zein proteins were fused together to create a new recombinant protein, zeolin, characterized by a balanced amino acid content. The chimeric zeolin protein contained the entire phaseolin sequence, including the signal peptide, followed by the unstructured 15 amino acid linker (GGGGS)_3_ and 89 amino acids of mature γ-zein (27 kDa), starting from the fifth residue after the γ-zein signal peptide. The total number of amino acids was 525, including 24 residues of the N-terminal phaseolin signal peptide (Mainieri et al., 2004). When expressed in *Nicotiana tabacum* leaves, zeolin successfully accumulated, forming PBs in the ER (Mainieri et al., 2004). Zeolin is insoluble in the absence of reducing agents, whereas, phaseolin can be easily solubilized in the absence of reducing agents. This suggests that the protein interactions are different. Insolubility is also caused by disulfide bonds, a characteristic of γ-zein (Vitale et al., 1982) which are transferred to zeolin. It was also observed that zein has a dominant effect on phaseolin intracellular traffic: the zein fragment prevents zeolin from being delivered to the vacuole and ER retention can be conferred to another protein by γ-zein domains (Holding, 2014).

In this study, heterologous expression of zeolin was obtained in the green alga *C. reinhardtii* which could play an integral role in paving the way for the production of a microalgal superfood source. Zeolin gene, previously expressed in tobacco leaves (Mainieri et al., 2004), was here completely redesigned in order to optimize its expression in *C. reinhardtii*. Four different vectors were designed to express and accumulate the targeted or retained protein of interest in the endoplasmic reticulum of *C. reinhardtii*. The different expression vectors were characterized for having specific sequences at N and/or C terminus to achieve ER localization of zeolin: phaseolin or BIP signal peptide was used to target the protein inside the ER, while HDEL sequence was added as an ER retention sequence.

## 2 Materials and methods

### 2.1 Algal strains and culture conditions

*C. reinhardtii* UVM4 (UV-mediated mutant 4) (Neupert et al., 2009) strain was used as the background for all the transformations. Algal cells were cultivated under mixotrophic conditions using Tris-acetate-phosphate (TAP) (Kropat et al., 2011)in shaker flasks at 25°C and 100–150 μmol photons m^−2^ s^−1^ of continuous white light, unless otherwise stated. Cultivation on solidified agar plates was performed under the same conditions.

### 2.2 Construction of transformation vectors, transformation, and mutant screening

**Figure 1:**
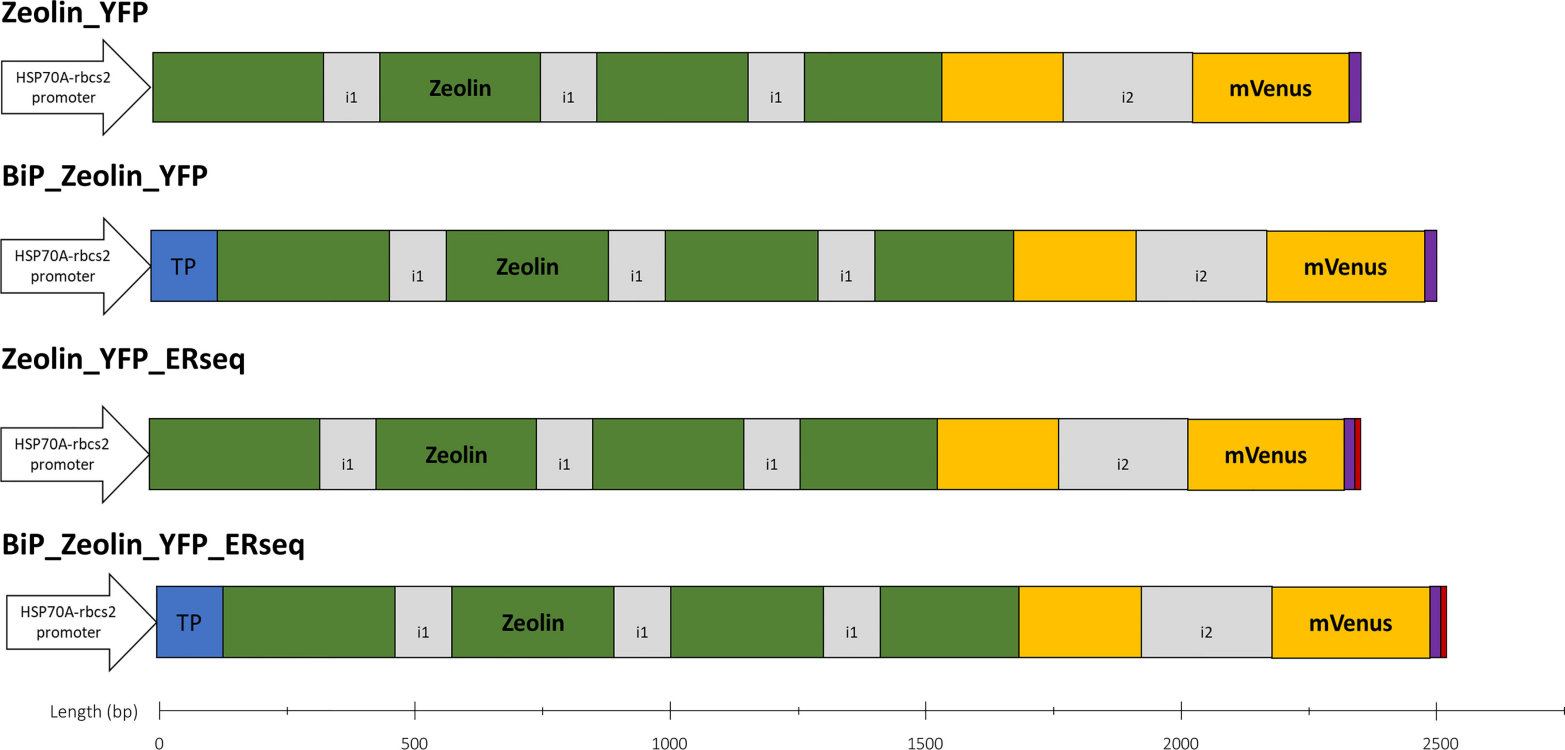
Zeolin expression vectors. Schematic overview of all expression vectors used in this work. All expression cassettes use the HSP70A-Rbcs2 hybrid promoter (containing *rbcs2* intron 1 and its 5′ UTR), and coding sequence are in frame with mVenus (YFP) sequence to generate a fusion protein. BIP1 target peptide (blue) directs proteins into ER. HDEL sequence (red) is used to retain proteins into ER. All proteins expressed carry a 10x H-tag at the C-terminus (purple). Intron 1 (i1) and 2 (i2) of *rbcs2* are depicted in grey color.

The zeolin-expressing vectors for *C. reinhardtii* transformation were prepared as follows. Starting from the amino acid sequence of zeolin (**Supplementary Figure S1**), the nucleotide sequence was optimized in silico, considering *C. reinhardtii* codon usage using Optimizer online tool (Puigbò et al., 2007). The mVenus (YFP) fluorescent coding sequence was added at the C-terminus of zeolin as an expression reporter for the selection of expression lines. A GSG-linker was also added between zeolin and YFP to allow the correct folding of the two proteins. To enhance protein expression and accumulation, RuBisCO introns were added to the coding sequence. Three *rbcs2* intron 1 copies were inserted into the zeolin sequence, and *rbcs2* intron 2 was added to the mVenus sequence according to the protocol designed by Baier et al. (Baier et al., 2018b). The optimized synthetic zeolin sequence is shown in **Supplementary Figure S2**. Additional peptides were then added at the N- or C-terminus to drive intracellular localization of zeolin: N terminus of the *C. reinhardtii* BiP1 protein (A8I7T8, herein named BiP) was used to drive ER localization, while HDEL ER retention sequence was added at the C-terminus (Rasala et al., 2014) as reported in the schematic diagram of the different vectors used in **Figure 1**.

The synthesized nucleotide sequence (Thermo Scientific, USA) was BamHI-BglII cloned into a pOpt2 modified vector. This vector was modified from the original pOpt2 (Lauersen et al., 2015) containing a 10x H-tag instead of S-tag. Conversely, BIP sequence was amplified with specific primer from pCM0-056 vector included in MocloToolkit (Crozet et al., 2018) and NdeI-BamHI cloned into pOpt2_Zeolin_YFP_Paro vector while the HDEL sequence was inserted into pOpt2_Zeolin_YFP_Paro and pOpt2_BIP_Zeolin_YFP_Paro by amplification of YFP with overhang primers and subsequent BglII-EcoRI cloning.

The different vectors were then used to transform the microalga *C. reinhardtii*; the UVM4 mutant strain, previously selected for efficient heterologous protein transformation, was adopted as the background (Neupert et al., 2009). Stable nuclear transformation was performed by glass beads agitation as previously described (Kindle, 1990). Transformants were selected on TAP agar plates supplied with 12 μg/mL of paromomycin for 6–7 days at a light intensity of 200 μmol photons m^−2^ s^−1^. Antibiotic-resistant colonies were cultivated in 96–well microtiter plates at a light intensity of 200 μmol photons m^−2^ s^−1^ until they were sufficiently dense. YFP fluorescence was measured using an Infinite PRO 200 plate reader (TECAN, Switzerland) with excitation at 509 ± 4.5 nm and emission at 540 ± 10 nm. Signals were normalized to 720 nm absorbance (cell scattering) to determine the highest expressing lines (Pivato et al., 2021).

### 2.3 Total protein extraction, SDS-PAGE, and western blotting

Total cells, exhaust growth media, and purified proteins were separated using SDS-PAGE (Laemmli, 1970). Separated proteins were stained using Coomassie Brilliant Blue solution or analyzed by immunodetection using an anti-GFP (Green Fluorescent Protein) antibody (Agrisera, Sweden). Protein accumulation over time in cells or supernatants was assessed by loading the same number of cells and the equivalent volume of growth medium obtained after centrifugation to remove cells followed by concentration using membrane spin columns (GE Healthcare, USA). The cell density was measured using Countess 3 (Thermo Scientific, USA). Protein quantification was performed by densitometric analysis using ImageLab software and recombinant YFP produced in *E.coli* as standard.

### 2.4 Zeolin purification

Zeolin_YFP was purified from BIP_Zeolin_YFP expressing lines, exploiting the presence of the H-tag at the C-terminus of the recombinant protein. After 4 days of cultivation, 1 L of culture was subjected to centrifugation to remove the cells, and the resulting supernatant containing secreted Zeolin_YFP was loaded onto an H-tag chromatographic affinity column. The elution was performed using 500 mM imidazole.

### 2.5 Growth analysis

Cell density was measured at 720 nm OD, and total dry biomass was evaluated by overnight lyophilization pellets followed by gravimetric determination as previously reported (Pivato et al., 2021). Cell dimension was measured by software analysis on microscope photos with n>70 (Perozeni et al., 2018). Statistical analysis was performed using a two-tailed t-test and compared with the UVM4.

### 2.6 Confocal microscopy

The subcellular localization of Zeolin_YFP was examined by confocal microscopy. Images were recorded using a Leica TCS-SP5 inverted confocal microscope (Leica Microsystems, Germany). mVenus (YFP) and chlorophyll were excited at 514 nm, and fluorescence emissions were detected at 522–572 nm and 680–720 nm for YFP and chlorophyll a, respectively as previously reported (Pivato et al., 2021).

## 3 Results

### 3.1 Zeolin expression in *Chlamydomonas reinhardtii*

Zeolin expression in *C. reinhardtii* was designed starting from the amino acid sequence previously expressed in tobacco (Mainieri et al., 2004) obtained by fusing T343 phaseolin from *Phaseolus vulgaris* L., including its signal peptide, with an unstructured 15 amino acids linker and 89 amino acids of mature γ-zein from *Zea mays* (**Supplementary Figure S1**). Zeolin gene for heterologous expression in *C. reinhardtii* was synthetically redesigned by codon optimization and intron spreading to enhance transgene expression, as previously described (Baier et al., 2018b) and reported in detailed in the Materials and Methods section. Previous work demonstrated that the highest zeolin accumulation in tobacco leaves was obtained when the protein accumulated in the ER while chloroplast localization was not efficient; therefore, chloroplast localization was not considered in this study. Instead, ER was targeted for intracellular zeolin localization. The zeolin sequence used in this study included a phaseolin N-terminus signal peptide, which was reported in tobacco to drive protein accumulation in the ER (expression vector Zeolin_YFP). To ensure a higher probability of the protein being targeted into the ER, a vector with an N-terminus signal peptide of the HSP70 molecular chaperone BiP (BiP1) from *C. reinhardtii* (Rasala et al., 2014) was also prepared (expression vector BiP_Zeolin_YFP). Finally, because successful zeolin protein expression in *Nicotiana tabacum* was obtained when zeolin was retained in the ER-forming protein bodies, expression vectors containing an ER retention sequence at the C-terminus of zeolin were obtained using the HDEL sequence previously reported to improve the retention of proteins in this compartment in *C. reinhardtii* (Rasala et al., 2014). HDEL sequences were added at the C-terminus of zeolin either in presence (BiP_Zeolin_YFP_Erseq expression vector) or absence (Zeolin_YFP_Erseq expression vector) of BiP signal sequence at N-terminus. A scheme of the different vectors adopted in this study is shown in **Figure 1**. The different vectors were then used to transform the microalga *C. reinhardtii*; the UVM4 mutant strain, previously selected for efficient heterologous protein transformation, was adopted as the background (Neupert et al., 2009).

**Figure 2:**
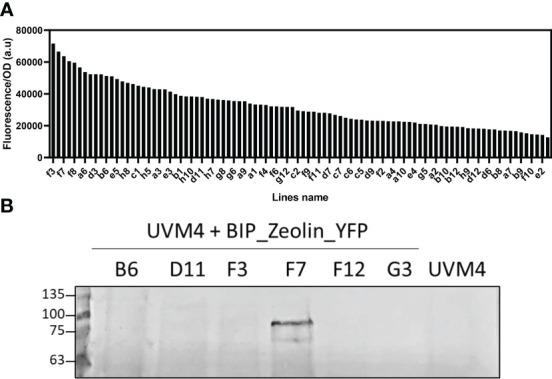
Fluorescence screening and Western blot analysis. **(A)** Fluorescence screening on putative BIP_Zeolin_YFP expressing lines (excitation: 509 ± 4.5 nm; emission: 540 ± 10 nm). **(B)** Western blot against YFP on putative BIP_Zeolin_YFP expressing lines (expected MW 85.5 kDa).

Putative transformant lines with the highest YFP fluorescence (at least 2-fold compared to the average YFP fluorescence emission of the screened lines) were then selected and investigated by western blotting (**Figure 2**). As reported in **Figure 2** and **Supplementary Figures S3–S5**, positive signals at ~80 kDa were identified for all the expression vectors used (**Table 1**). Considering the expected molecular weight of ~85.5 kDa for the mature zeolin-YFP protein, the slightly lower molecular weight observed could be related to additional proteolysis of the protein. The lines with the strongest accumulation of zeolin-YFP complex (D2.2 for Zeolin_YFP, F7 for BiP_Zeolin_YFP, E9.1, Zeolin_YFP_ER and B5.1 for BiP_Zeolin_YFP_ER, respectively) were then used for the following analysis.

**Table 1:** Numbers of lines screened by YFP fluorescence and western blotting.

Expression vector	Transformant screened	YFP positive lines	Lines selected (YFP fluorescence)	Zeolin-YFP expressing lines (western blot)
**Zeolin_YFP**	192	45	8	5
**BiP_Zeolin_YFP**	96	39	6	1
**Zeolin_YFP_ERseq_**	192	20	5	5
**BiP_Zeolin_YFP_ERseq**	168	83	5	1

‘YFP positive lines’ refers to the transformant lines with an increase in YFP fluorescence of at least two-fold after normalization to the cell scattering at 720 nm, compared to the average of the 20 lines with the lowest YFP fluorescence emission per 720-nm cell scattering. The lines selected after YFP screening are those whose YFP fluorescence showed an increase of at least 3-fold compared to the average of the 20 lines with the lowest YFP fluorescence emission per 720-nm cell scattering.

### 3.2 Zeolin localization

**Figure 3:**
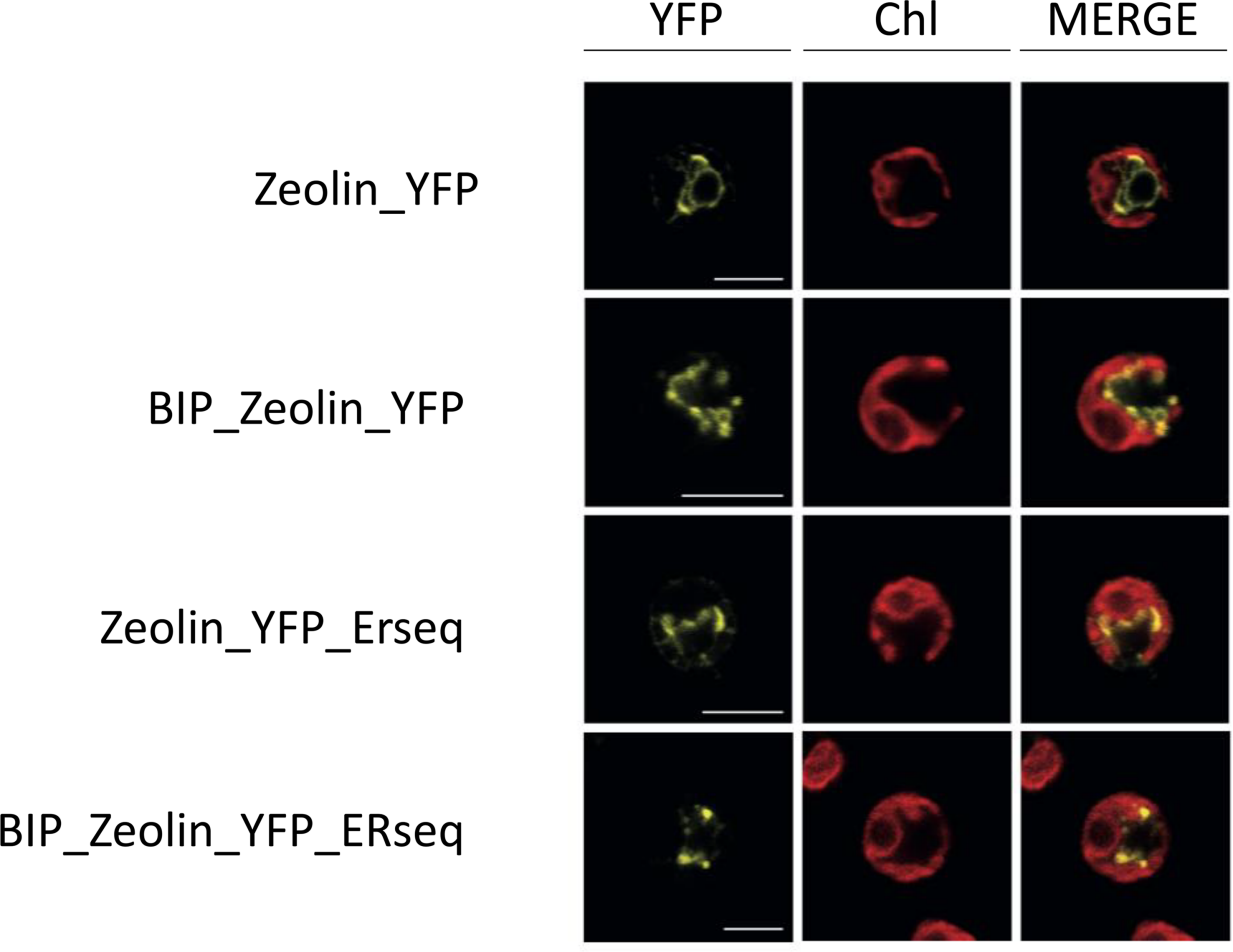
Zeolin localization. Analysis of endogenous (first line) or additional (lines 2–5) Zeolin_YFP target peptide. YFP fluorescence (YFP), chlorophyll autofluorescence (Chl), and merger of these two channels are shown. Excitation for YFP was 514 nm and 633 nm for chlorophylls. Emission was detected at 522–572 nm for YFP and 670–690 nm for chlorophylls. Scale bar represents 5 µm.

The presence of YFP fused to zeolin allowed the investigation of the zeolin-YFP complex localization by confocal microscopy (**Figure 3**). In the case of cells transformed with the Zeolin_YFP vector, the YFP signal was measured around the nucleus and did not overlap with the chlorophyll fluorescence signal (second column), which can be reconducted to ER localization according to literature (Rasala et al., 2014). The same behavior was observed in BiP_Zeolin_ YFP-expressing lines, demonstrating that either phaseolin or BiP N-terminus signal peptides drive zeolin translation in the ER. In the case of Zeolin_YFP_ERseq, where the ER retention sequence HDEL was added at the C-terminus of the zeolin-YFP complex, the YFP fluorescence signal was again detected as a net around the nucleus, consisting of ER localization and excluding nucleus, cytosol, or chloroplast localization. However, in some cases, the zeolin-YFP complex in Zeolin_YFP_ERseq transformant lines was found to be located in defined spherical structures with extremely high fluorescence, which can be reconducted to protein bodies, and the formation of protein bodies was reported in tobacco leaves to significantly increase the intracellular accumulation of zeolin (Mainieri et al., 2004). The variability observed in terms of protein body formation in Zeolin_YFP_ ERseq-expressing cells could be related to a different level of expression, with protein body formation where zeolin-YFP expression reached a certain level. Finally, the addition of both the BIP target peptide and the HDEL retention sequence gives confocal microscopy a hybrid picture. Proteins are both located in filaments around the nucleus but also in high-fluorescence circular bodies.

### 3.3 Zeolin influence on cell growth

**Figure 4:**
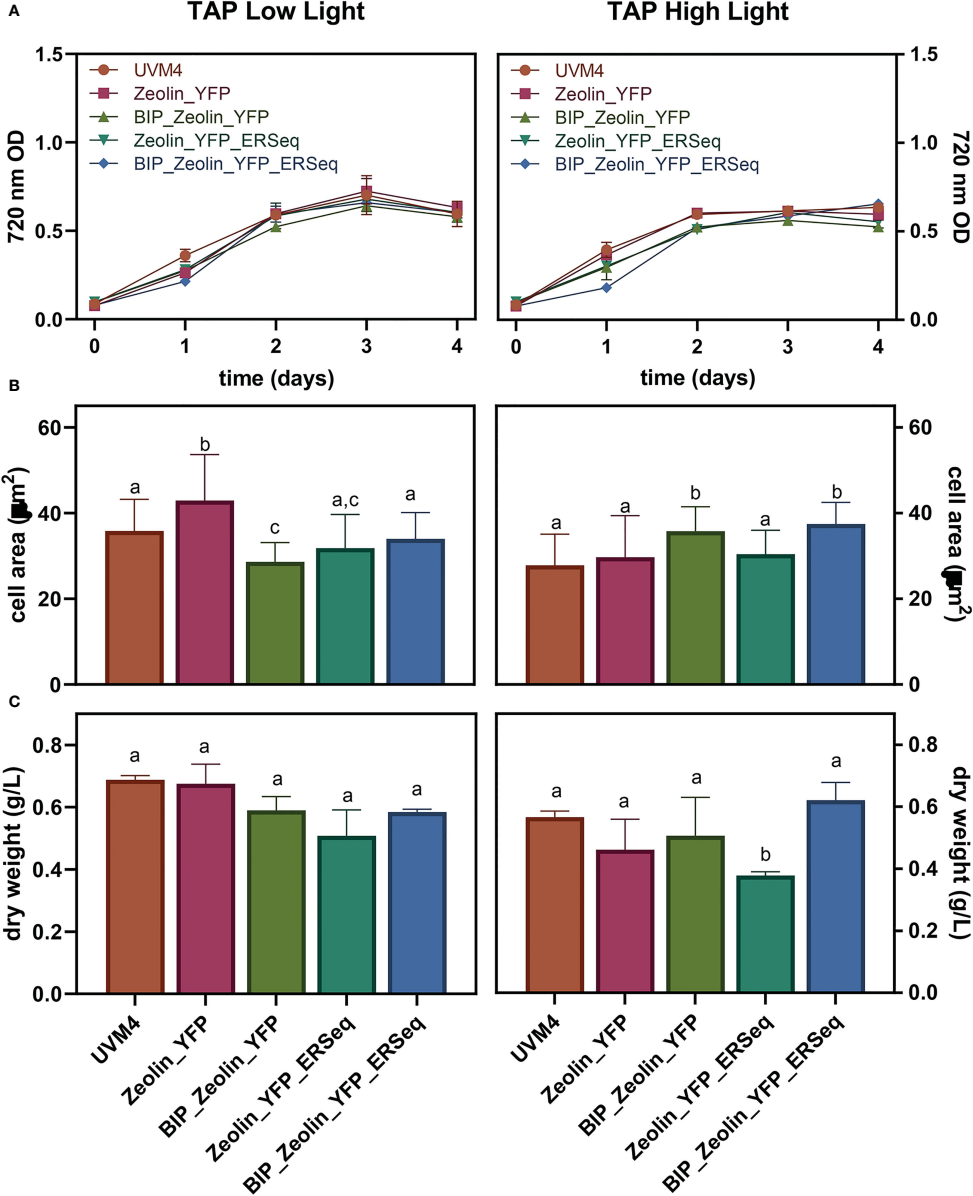
Expressing lines growth performance. Growth test was conducted in mixotrophy (TAP) in low (80 μmol photons m-2 s-2) or high (500 μmol photons m-2 s-2) light. 720 nm optical density **(A)**, cell dimensions **(B)** as well as dry biomass **(C)** were evaluated. The significantly different values (P < 0.05) in panels B and C are indicated with different letters. For cell areas **(B)** n=70 while for dry weight values **(C)** n=3.

The influence of zeolin-YFP expression on cell growth and biomass productivity was analyzed by cultivating the transformed lines in mixotrophic conditions under high (500 µmol m^-s^s^-1^) or low light (80 µmol m^-s^s^-1^) conditions. Cell scattering at 720 nm was used to follow the growth kinetics of the zeolin-expressing lines and their background UVM4. As shown in **Figure 4**, similar growth kinetics were observed for cells grown under low or high light conditions. Under either low or high light conditions, a slightly reduced growth could be observed for zeolin-expressing lines compared to UVM4 on the first days of cultivation, but on the second day under low light or on the third day under high light, no significant difference could be observed between transformed lines and UVM4. The cell areas of the different lines grown at 80 or 500 µmol m^-s^s^-1^ were then measured. Similar values between the different genotypes were retrieved and investigated for cells grown under high or low light. Biomass dry weight was also measured at the end of the growth curves, as shown in **Figure 4** Under high light conditions, increased biomass production was observed compared to low light conditions, likely a consequence of the increased light energy available. Under both low and high light conditions, the dry weights of the different zeolin-expressing lines were similar to those of UVM4, except for the Zeolin_YFP_ERseq transformant line, which was characterized by a slightly reduced dry weight compared to its background under both light conditions. Interestingly, the Zeolin_YFP_ERseq vector was the best condition for inducing zeolin accumulation in protein bodies, which could potentially have a minor negative effect on cell growth.

### 3.4 Zeolin is retained in ER only in the presence of HDEL sequence

**Figure 5:**
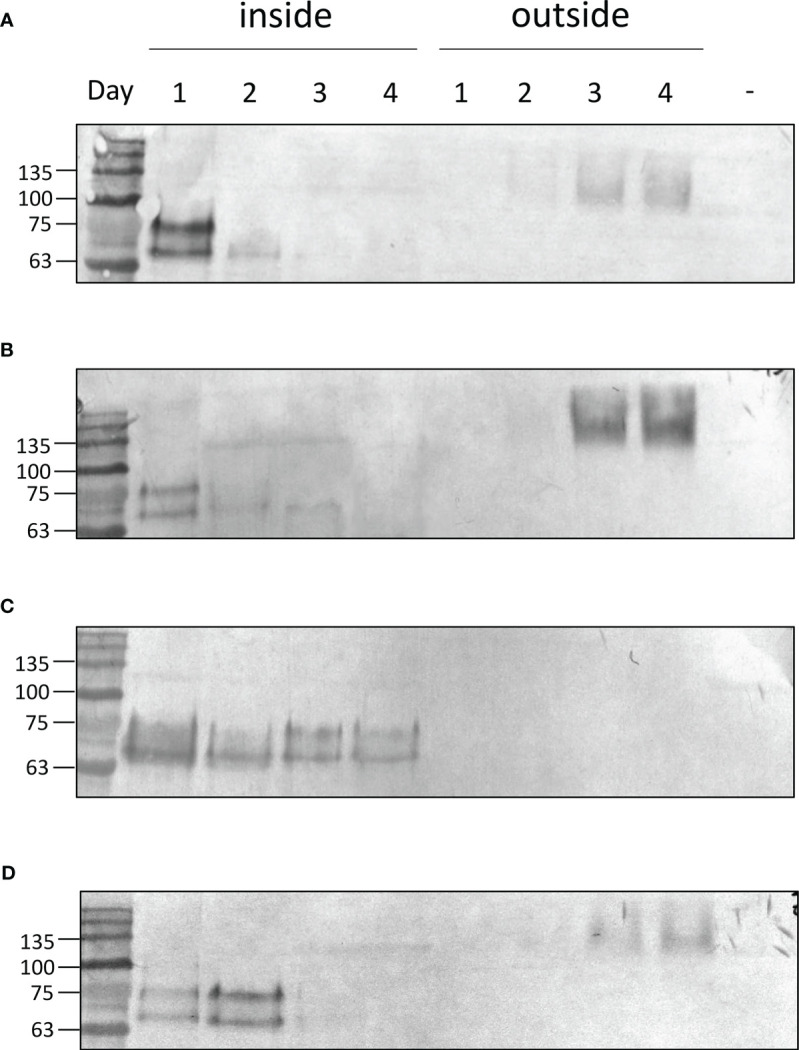
Zeolin accumulation and distribution. Western blot analysis showing protein accumulation at different days of cultivation in expressing lines either in the cell (inside) or secreted in the supernatant (outside). Data are referred to Zeolin_YFP **(A)**, BIP_Zeolin_YFP **(B)**, Zeolin_YFP_Erseq **(C)** and BIP_Zeolin_YFP_Erseq **(D)**. The negative control is marked with **(-)** and is represented by UVM4 supernatant at day 4.

Zeolin is retained in the ER of plant leaves. In the case of *C. reinhardtii*, proteins targeted to the ER were reported to be secreted in several locations and loaded in vesicles targeted to the plasma membrane. To monitor zeolin secretion in the expressing lines, western blot analysis of cells and growth media was performed at different times of cultivation. UVM4 and zeolin-expressing lines were cultivated for up to 4 d in TAP medium at 200 µmol m^-s^s^-1^. At different cultivation times, cells were harvested by centrifugation, and pellets and supernatants were separately analyzed by western blotting using α-GFP antibody recognizing the zeolin-YFP complex. In the case of UVM4, no YFP bands were detected in cells or in the exhausted growth medium during the four days of sampling, as expected (**Supplementary Figure S6**). In contrast, as shown in **Figure 5**, for the Zeolin_YFP transformed line, a double band at ~80 kDa was detected in cells harvested after 1 d of cultivation, which strongly decreased in the following days of cultivation, accompanied by an increase in the zeolin-YFP signal at higher molecular weight (~140 kDa) appearing in the supernatant, suggesting a possible secretion in dimeric form (see below for further details). The observation of a double band of the zeolin-YFP complex suggests the presence of partial protein degradation, as previously observed in the case of zeolin expressed in tobacco leaves (Mainieri et al., 2004), whereas the appearance of a clear band in the supernatants suggests zeolin-YFP protein secretion. Similar results were obtained for BiP_Zeolin_YFP, where most of the zeolin-YFP complexes were present in the supernatant after 3 or 4 d of cultivation. Completely different results were obtained in the case of Zeolin_YFP_ERseq transformant lines; in this case, YFP positive signals as a double band at ~80 kDa were observed only in cells for all four days of cultivation, while no zeolin-YFP could be detected in the growth medium. This result suggests that zeolin-YFP was successfully retained inside the cell and was not secreted in the presence of the HDEL sequence. In the case of the BIP_Zeolin_YFP_ERseq line, where both BiP and HDEL sequences were added to the N- and C-termini of the zeolin-YFP complex, a double band was detected at 80 kDa in the pellet samples, with the strongest protein signal observed on the second day in the pellet, whereas in the supernatant, a clear band appeared only on the fourth day. These results suggest that in the presence of both the BiP signal peptide and HDEL ER retention signal, the secretion of the protein is present yet delayed compared to when the HDEL sequence is absent (Zeolin_YFP and BiP_Zeolin_YFP). A possible explanation could be that the double signal peptide (the one contained in the zeolin sequence and the extra one from Bip) delays the folding of the protein, which is therefore retained in the ER for longer periods before being secreted, resulting in an intermediate phenotype compared to the case in which only one of the two sequences was added to the zeolin-YFP complex.

The zeolin-YFP complex secreted in the expression lines Zeolin_YFP, BiP_Zeoin_YFP, and BIP_Zeolin_YFP_ERseq was detected at a higher molecular weight than the zeolin-YFP complex retained in the cells (**Figure 5**). The higher molecular weight of the secreted protein can be caused by a post-translational modification (i.e., N-glycosylation, as in phaseolin) or by interference in the electrophoretic pathway, caused by the salts contained in the growth medium or by the formation of complex aggregates in the secreted proteins. Another possibility is that the protein forms a dimer once it reaches the extracellular space, which would explain the molecular weight of approximately 150 kDa, which is exactly double the weight of the protein found inside the cells. To investigate the latter hypothesis, the secreted zeolin_YFP was purified from the growth medium by affinity chromatography owing to the presence of a His tag at the C-terminus. The purified zeolin-YFP was studied by western blot analysis. As shown in **Supplementary Figure S7**, a band of approximately 150 kDa was detected with the YFP antibody; these data are consistent with what was previously observed in western blot analysis of the supernatant. To clarify the nature of the 150 kDa band, western blot analysis was repeated by incubating the purified Zeolin-YFP complex at 100°C for 5 min to interrupt possible states of aggregation. Heat treatment resulted in a decrease in the molecular weight of the protein, while the intensity of the band remained the same, possibly indicating that the higher apparent molecular weight migration was due to the formation of zeolin-YFP dimers.

**Figure 6:**
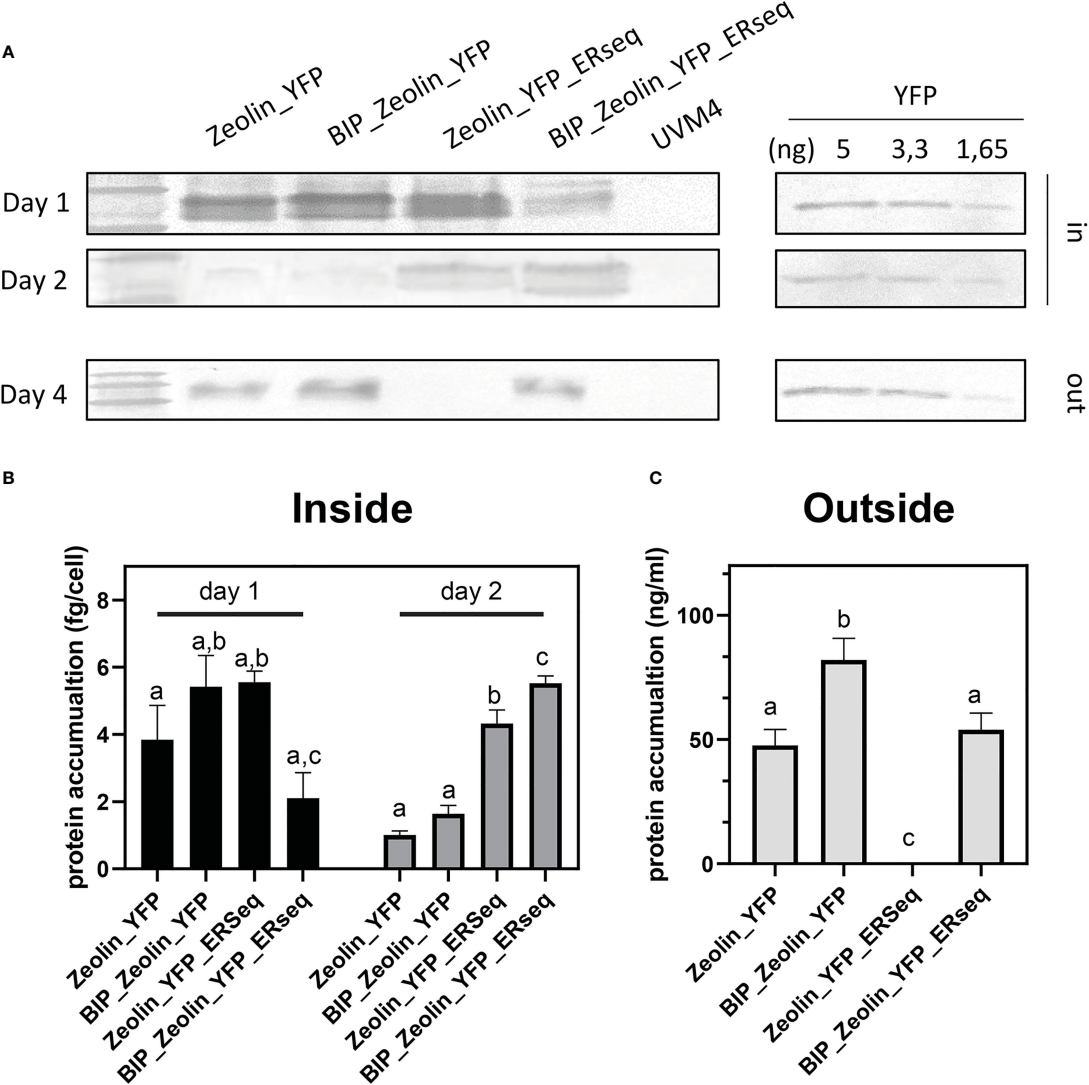
Zeolin quantification. **(A)** Quantitative western blot of Zeolin accumulating lines both for cell (inside) and supernatant (outside). YFP used as standard is shown with the loaded amount. **(B)** Zeolin quantification inside cell for zeolin expressing lines at day 1 and 2. **(C)** Zeolin quantification into supernatant (outside) for zeolin expressing lines at day 4. Results represent mean values and standard deviation from biological triplicates (n = 3). The significantly differences (P < 0.05) between different genotype at the same time point and same localization (inside or outside the cells) are indicated with different letters.

The quantification of the zeolin accumulated in the cells or secreted into the medium is reported in **Figure 6** and was calculated using isolated YFP as a standard. The maximum zeolin accumulation per cell was observed in the Zeolin_YFP_ERseq expressing line after two days of cultivation. The reduced content on the fourth day suggested that zeolin protein bodies accumulated in the Zeolin_YFP_ Erseq-expressing line were partially degraded and/or their biosynthesis was reduced during cell cultivation. Finally, the total protein content in the different zeolin-expressing lines was analyzed, as reported in **Table 2**. Similar total protein content was measured in all the different zeolin-expressing lines compared to the UVM4 background, even though a slight increase in the total protein fraction per cell could be observed in the case of Zeolin_YFP_Erseq compared to Zeolin_YFP and BiP_Zeoin_YFP transformant lines.

**Table 2:** Protein content per dry weight of zeolin-YFP expressing lines.

	UVM4	Zeolin_YFP	BiP_Zeolin_YFP	Zeolin_YFP_ERseq_	BiP_Zeolin_YFP_ERseq
**% protein/total weight**	30.9 ± 4.7 ^a,b^	23.8 ± 2.8 ^b^	24.5 ± 2.0 ^b^	35.9 ± 6.1 ^a,c^	29.1 ± 1.1 ^a,c^

Total protein content in the different zeolin-expressing lines measured using micro BCA assay kit (Thermo Fisher). The significantly different values (P< 0.05) are indicated with different letters.

## 4 Discussion

Seed storage proteins in legumes and cereals are the two main sources of protein for human nutrition. However, seed storage proteins from legumes are poor in sulfur amino acids, whereas cereal proteins are poor in Lysine and Tryptophan. Zeolin, the chimeric protein obtained by fusing phaseolin and zein, was proposed as a possible solution to provide a complete and balanced amino acid nutrition profile (Mainieri et al., 2004). In this study, we obtained heterologous expression of zeolin in the model organism for the green alga *C. reinhardtii*, which can be considered a novel sustainable protein source, with its ability to use light energy to assimilate CO_2_ and inorganic nutrients into edible biomass without competing for land and water with other food crops. Moreover, the high photosynthetic efficiency of microalgae and their lower water footprint compared to crops make these organisms potential candidates to support life during space exploration as food and oxygen providers. The protein content of microalgae is species-related, but typically is very high, ranging from 30 to 60% of the total dry matter (Wang et al., 2021). The goal of this study was to further modify the protein content by introducing a storage protein with balanced amino acids, which could potentially accumulate in protein bodies. Zeolin localization in the ER was targeted because this compartment is a suitable organelle for protein accumulation, and previous studies in tobacco have shown that zeolin reaches a high concentration in the ER-forming inclusion body.

Zeolin_YFP-expressing lines were stable and showed protein accumulation in the ER at the initial stage of cultivation, whereas zeolin-YFP secretion was observed in the mid-exponential and saturation phases. These results demonstrate that the N-terminus phaseolin signal peptide is recognized as an entry signal to the secretory pathway in *C. reinhardtii*. The secretion of zeolin-YFP further support the transient ER localization of the protein, being the translation in the ER required for the secretion of heterologous proteins in *C. reinhardtii* (Lauersen et al., 2013; Rasala et al., 2014; Baier et al., 2018a; Molino et al., 2018). Zeolin-YFP secretion is likely at the base of some discrepancy observed between YFP screening and western blot analysis of transformant lines (**Figure 2**; **Supplementary Figures S3–5**) because the cells were not synchronized for this screening procedure. In *Nicotiana tabacum*, zeolin forms protein bodies in the ER; however, considering that the two hosts have two different cell structures, it is reasonable that the mechanism is not conserved. The secretion of zeolin-YFP in the engineered strains described herein demonstrates that the mechanism of ER retention is different in zeolin-expressing tobacco leaves and *C. reinhardtii* cells. Alternatively, it is possible that YFP at the C-terminus of the recombinant zeolin-YFP complex negatively affects ER retention and protein body formation. HDEL addition at the C-terminus led to retention of the protein inside the ER. Even if we cannot exclude the possibility that a fraction of zeolin may be confined in the cytosol, interfering with ER import. ER retention mediated by the HDEL sequence caused the formation of protein bodies, which were clearly visible by confocal microscopy. As previously suggested, zeolin protein bodies are likely too large to be packed in vesicles entering the secretion pathway (Mainieri et al., 2004). The simultaneous addition of the BiP signal peptide at the N-terminus and HDEL at the C-terminus led to an intermediate phenotype, with most zeolin-YFP retained in the cells even after two days of cultivation, whereas at the saturation phase (days 3 and 4), the chimeric protein was essentially secreted. According to these results, the presence of the BIP transit peptide strongly improved the ER localization of zeolin-YFP and its delivery in the secretion pathway, while the HDEL sequence at the C-terminus increased its retention in the ER. It is important to note that in plants, both KDEL and HDEL ER retention signals also promote protein delivery into the vacuole (Gomord et al., 1997): In the case of *C. reinhardtii*, HDEL sequence was reported to induce ER retention, but we cannot exclude that zeolin-YFP with the HDEL sequence at the C-terminus might be partially delivered to other hydrolytic compartments in *C. reinhardtii* cells. The slightly reduced biomass accumulation observed in Zeolin_YFP_ERseq expressing lines raises the question of a possible negative effect on growth due to protein body formation in the ER, which might induce the onset of protein degradation pathways.

The Zeolin_YFP retained in the cell was detected by SDS-PAGE as a double band at ~80kDa. The expected molecular weight of the mature zeolin-YFP complex is 85.5 kDa: the lower apparent molecular weight observed may be due to partial proteolysis of the protein. It is interesting to note that when retained in the cell, the protein was detected as a double band, as reported by Mainieri et. al., probably referring to the presence of two different proteolytic products. The secreted zeolin-YFP chimeric protein was detected at a higher molecular weight (~140 kDa) due to the presence of protein aggregates (dimers) that can be dissolved upon thermal treatment (**Supplementary Figure S7**). The nature of zein can explain its higher molecular weight; zein appears as a heterologous zein mix (disulfide-linked aggregates), in which γ-zein is the starting point and is essential for protein body formation. Moreover, post-translational modifications are likely to occur, probably glycosylation, considering that in beans, phaseolin monomers are N-glycosylated and transported from the ER and Golgi complex to the protein storage vacuoles.

Zeolin production yield by engineered strains was quite low, reaching values of ~6 fg/cell when retained in the ER, whereas a concentration of ~82 µg/L was obtained when zeolin-YFP was secreted. Several reasons can explain why the production is low. A negative effect could be related to the presence of YFP and His tags and the C-terminus; it is well known that different tags could have different impacts on protein expression in both prokaryotic and eukaryotic cells (Baier et al., 2018a; Koppl et al., 2022). Moreover, we cannot exclude the possibility that the protein could accumulate and degrade without forming protein bodies. It is important to note that zeolin retention in the ER is facilitated by the formation of Cys-bound in its C-terminus region. In our zeolin-YFP chimeric protein, the C-terminus contains YFP, a 27-kDa protein with a well-defined secondary structure that can interfere with the formation of Cys bonds and thus, ER retention. A strategy to fully exploit the zeolin potential is represented by the expression of zeolite alone, without any fluorophore. With the exception of the zeolin-YFP variant presenting the HDEL sequence at the C-terminus, the recombinant chimeric protein was secreted, excluding possible feedback inhibition due to protein accumulation. A further possible explanation could be the correlation between zeolin-YFP production and acetate availability (carbon source in TAP medium), indicating that depletion of the carbon source in the first two days causes a decrease in zeolin-YFP biosynthesis. To study this hypothesis and test C level as a limiting factor, further experiments must be performed under autotrophy conditions (CO_2_ as a C source) or constant acetate supplementation. In addition to the random nature of gene insertion, expression is affected by the position effect; thus, by screening a large number of lines, it is possible to obtain lines with higher accumulation. Moreover, insertion of multiple gene copies could have a positive effect on protein accumulation. Finally, we cannot exclude the possibility that other growth conditions may have different effects on protein production and accumulation.

In conclusion, we obtained heterologous expression of a chimeric seed storage protein in a model organism for green alga, *C. reinhardtii*. Using the ER retention sequence HDEL, it was possible to induce zeolin accumulation as protein bodies in the ER, even with low expression efficiency. It is interesting to note that *C. reinhardtii* has already been recognized as safe for human consumption by the FDA, and previous work has demonstrated the possibility of inducing the accumulation of other compounds important for human nutrition, such as antioxidants or omega-3 fatty acids (Nguyen et al., 2013). With the objective of obtaining a sustainable superfood with a high nutritional profile and the ability to convert CO_2_ and inorganic nutrients into edible biomass, zeolin expression in *C. reinhardtii* combined with metabolic engineering represents a possible solution.

## Data availability statement

The original contributions presented in the study are included in the article/**Supplementary Material**. Further inquiries can be directed to the corresponding author.

## Author contributions

MB and AP conceived of the study. MB and AP supervised experiments. FP and MA performed or contributed to all experiments reported herein. MP performed confocal microscopy analysis. MB, FP, and AP wrote the manuscript with contributions from all authors. All authors discussed the results, contributed to data interpretation, and commented on the manuscript. All authors contributed to the article and approved the submitted version.
