# Supplementary material for: Towards microalga-based superfoods: heterologous expression of zeolin in Chlamydomonas reinhardtii
Source: Front Plant Sci. 2023 May 9;14:1184064. doi: 10.3389/fpls.2023.1184064 (PMC10203602; doi:10.3389/fpls.2023.1184064)
Supplement: Supplementary file 1 [file DataSheet_1.pdf]

frontiers

Supplementary Material

Towards microalga-based superfoods: heterologous expression of zeolin in *Chlamydomonas reinhardtii*

Federico Perozeni[1], Matteo Pivato[1], Margherita Angelini[1], Elisa Maricchiolo[2], Andrea Pompa[2], Matteo Ballottari1[1*]

[1]Dipartimento di Biotecnologie, Università degli Studi di Verona,Verona, Italy

[2]Dipartimento di Scienze Biomolecolari, Università degli studi di Urbino, Urbino, Italy

* **Correspondence:**
Corresponding Author
matteo.ballottari@univr.it

1 Supplementary Figures and Tables

For more information on Supplementary Material and for details on the different file types accepted, please see here.

1.1 Supplementary Figures

```
MAQWKAAVLLLALACASYGFGVWAEEEKLGTVIGGSMRARVPLLLLGILFLASLSASFATSLREEEE
SQDNPFYFNSDNSWNTLFKNQYGHIRVLRFDQQSKRLQNLEDYRLVEFRSKPETLLLPQQADAELLL
VVRSGSAILVLVKPDDRREYFFLTSDNPIFSDHQKIPAGTIFYLVNPDPKEDLRIIQLAMVNNPQIH
EFFLSSTEAQQSYLQEFSKHILEASFNSKFEEINRVLFEEEGQEGVIVNIDSEQIKKLSKHAKSSSR
KSLSKQDNTIGNEFGNLTERTDNSLNVLISSIEMEEGALFVPHYYSKAIVILVVNEGEAHVELVGPK
GNKETLEYESYRAELSKDDVFVIPAAYPVAIKATSNVNFFGFGINANNNNRNLLAGKTDNVISSIGR
ALDGKDVLGLTFSGSGDEVMKLINKQSGSYFVDAHHHQQEQQKGRKGAFVYGGGGSGGGGSGGGGSG
GCGCQPPPPVHLPPPVHLPPPVHLPPPVHLPPPVHLPPPVHLPPPVHVPPPVHLPPPPCHYPTQPPR
PQPHPQPHPCPCQQPHPSPCQGSGRSDVVSKGEELFTGVVPILVELDGDVNGHKFSVSGEGEGDATY
GKLTLKLICTTGKLPVPWPTLVTTLGYGLQCFARYPDHMKQHDFFKSAMPEGYVQERTIFFKDDGNY
KTRAEVKFEGDTLVNRIELKGIDFKEDGNILGHKLEYNYNSHNVYITADKQKNGIKANFKIRHNIED
GGVQLADHYQQNTPIGDGPVLLPDNHYLSYQSKLSKDPNEKRDHMVLLEFVTAAGITLGMDELYKIE
GRDIEFHHHHHHHHHHHDEL*
```

Supplementary Figure 1. Zeolin protein sequence (black). BIP1 ER target peptide is blue written while endogenous target peptide is dark gray shaded. GSG linker (green), mVenus (orange), FactoXa cleavage site (purple), H-tag (red) and HDEL ER retention sequence are also highlighted.

CATATGGGATCCATGCGCGCCCGCGTGCCCCTGCTGCTGCTGGGCATCCTGTTCCTGGCCAGCCTGA
GCGCCAGCTTCGCCACCAGCCTGCGCGAGGAGGAGGAGAGCCAGGACAACCCCTTCTACTTCAACAG
CGACAACAGCTGGAACACCCTGTTCAAGAACCAGTACGGCCACATCCGCGTGCTGCGCTTCGACCAG
CAGAGCAAGCGCCTGCAGAACCTGGAGGACTACCGCCTGGTGGAGTTCCGCAGCAAGCCCGAGACCC
TGCTGCTGCCCCAGCAGGCCGACGCCGAGCTGCTGCTGGTGGTGCGCAGCGGCAGCGCCATCCTGGT
GCTGGTGAAGCCCGACGACCGCCGCGAGTACTTCTTCCTGACCAGCGACAACCCCATCTTCAGCGAC
CACCAGAAGATCCCCGCCGGCACCATCTTCTACCTGgtgagtcgacgagcaagcccggcggatcagg
cagcgtgcttgcagatttgacttgcaacgcccgcattgtgtcgacgaaggcttttggctcctctgtc
gctgtctcaagcagcatctaaccctgcgtcgccgtttccatttgcagGTGAACCCCGACCCCAAGGA
GGACCTGCGCATCATCCAGCTGGCCATGGTGAACAACCCCCAGATCCACGAGTTCTTCCTGAGCAGC
ACCGAGGCCCAGCAGAGCTACCTGCAGGAGTTCAGCAAGCACATCCTGGAGGCCAGCTTCAACAGCA
AGTTCGAGGAGATCAACCGCGTGCTGTTCGAGGAGGAGGGCCAGGAGGGCGTGATCGTGAACATCGA
CAGCGAGCAGATCAAGAAGCTGAGCAAGCACGCCAAGAGCAGCAGCCGCAAGAGCCTGAGCAAGCAG
GACAACACCATCGGCAACGAGTTCGGCAACCTGACCGAGCGCACCGACAACAGCCTGAACGTGCTGA
TCAGCAGCATCGAGATGGAGGAGGGCGCCCTGTTCGTGCCCCACTACTACAGCAAGgtgagtcgacg
agcaagcccggcggatcaggcagcgtgcttgcagatttgacttgcaacgcccgcattgtgtcgacga
aggcttttggctcctctgtcgctgtctcaagcagcatctaaccctgcgtcgccgtttccatttgcag
GCCATCGTGATCCTGGTGGTGAACGAGGGCGAGGCCCACGTGGAGCTGGTGGGCCCCAAGGGCAACA
AGGAGACCCTGGAGTACGAGAGCTACCGCGCCGAGCTGAGCAAGGACGACGTGTTCGTGATCCCCGC
CGCCTACCCCGTGGCCATCAAGGCCACCAGCAACGTGAACTTCTTCGGCTTCGGCATCAACGCCAAC
AACAACAACCGCAACCTGCTGGCCGGCAAGACCGACAACGTGATCAGCAGCATCGGCCGCGCCCTGG
ACGGCAAGGACGTGCTGGGCCTGACCTTCAGCGGCAGCGGCGACGAGGTGATGAAGCTGATCAACAA
GCAGAGCGGCAGCTACTTCGTGGACGCCCACCACCACCAGCAGGAGCAGCAGAAGgtgagtcgacga
gcaagcccggcggatcaggcagcgtgcttgcagatttgacttgcaacgcccgcattgtgtcgacgaa
ggcttttggctcctctgtcgctgtctcaagcagcatctaaccctgcgtcgccgtttccatttgcagG
GCCGCAAGGGCGCCTTCGTGTACGGCGGCGGCGGCAGCGGCGGCGGCGGCAGCGGCGGCGGCGGCAG
CGGCGGCTGCGGCTGCCAGCCCCCCCCCCCCGTGCACCTGCCCCCCCCCGTGCACCTGCCCCCCCCC
GTGCACCTGCCCCCCCCCGTGCACCTGCCCCCCCCCGTGCACCTGCCCCCCCCCGTGCACCTGCCCC
CCCCCGTGCACGTGCCCCCCCCCGTGCACCTGCCCCCCCCCCCCTGCCACTACCCCACCCAGCCCCC
CCGCCCCCAGCCCCACCCCCAGCCCCACCCCTGCCCCTGCCAGCAGCCCCACCCCAGCCCCTGCCAG
GGCAGCGGCAGATCT

Supplementary Figure 2. Optimized zeolin sequence. rbcs2 intron1 are highlight in orange while in red (BamHI), blue (NdeI), purple (BglII) restriction sites and GSG linker (green)

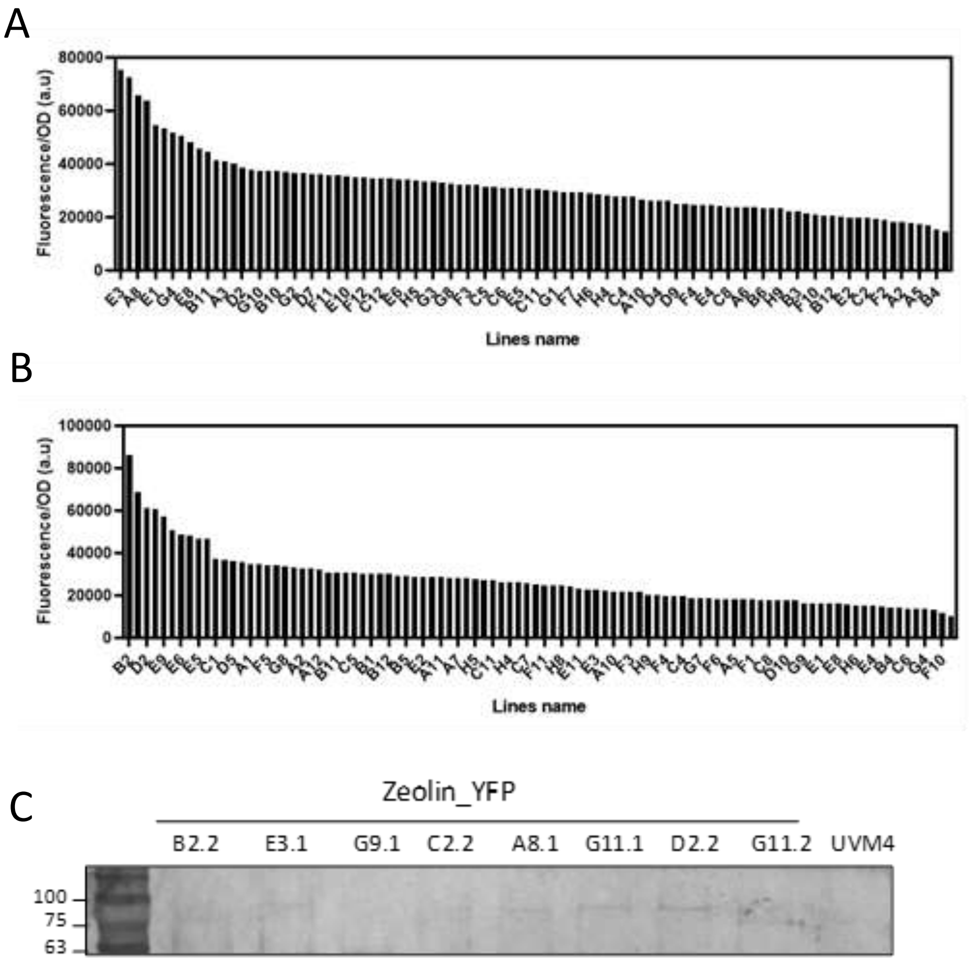

Supplementary Figure 3. YFP fluorescence screening (A) for 1-96 lines and (B) for 97-192. Western blot analysis of putative Zeolin_YFP transformant lines (C). UVM4 is reported as negative control.

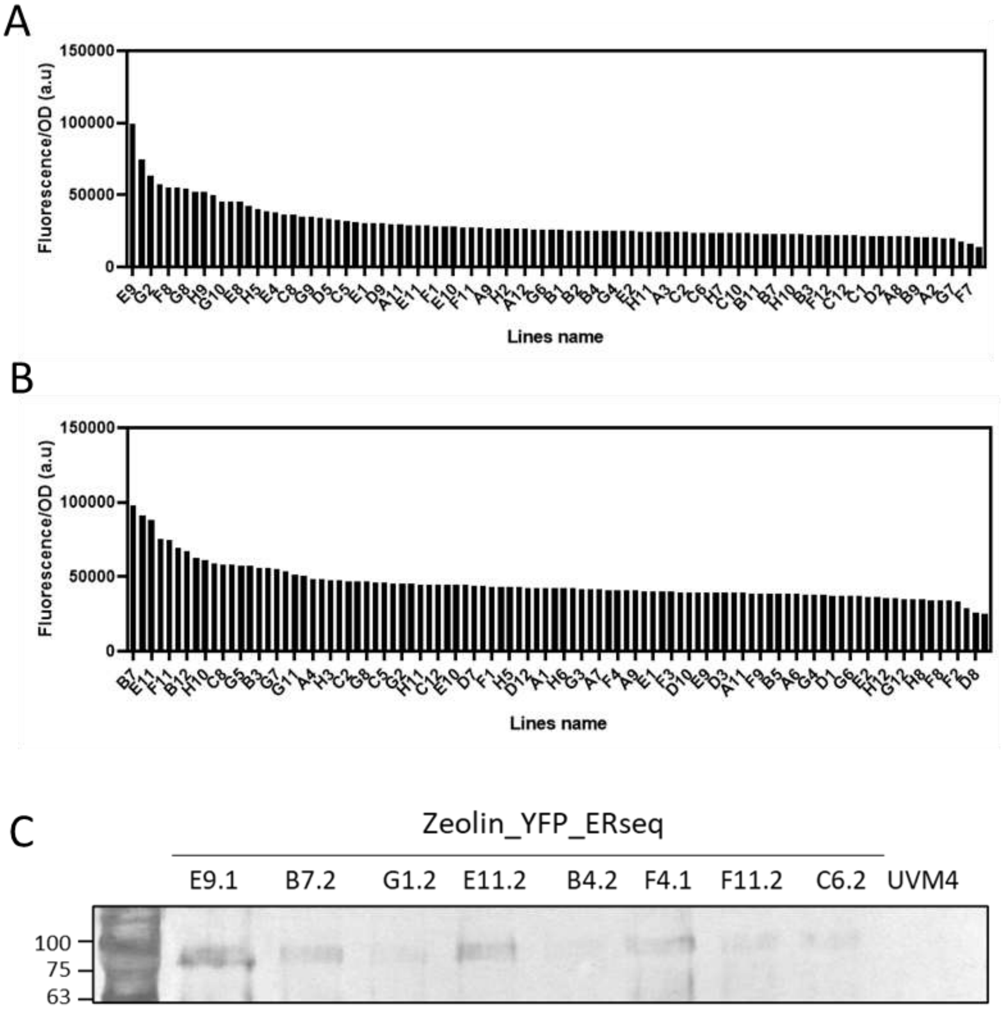

Supplementary Figure 4. YFP fluorescence screening and western blot analysis of putative Zeolin_YFP_ERseq transformant lines. UVM4 is reported as negative control.

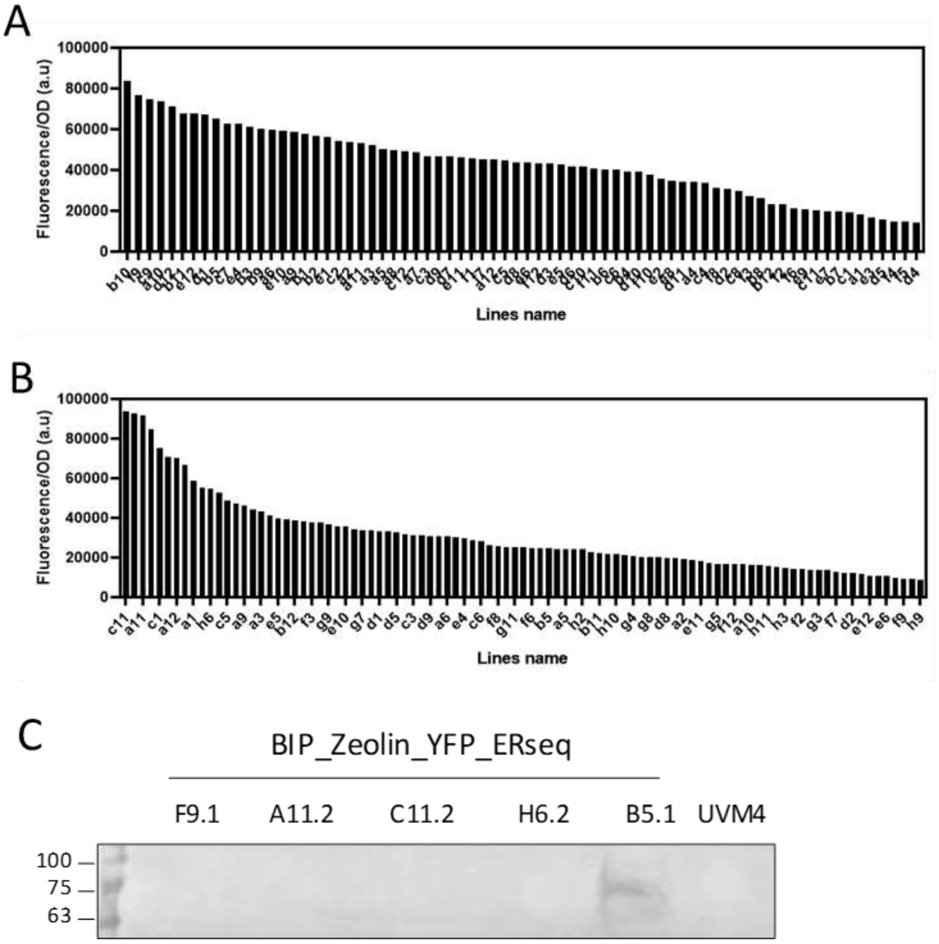

Supplementary Figure 5. YFP fluorescence screening and western blot analysis of putative BiP_Zeolin_YFP_ERseq transformant lines. UVM4 is reported as negative control.

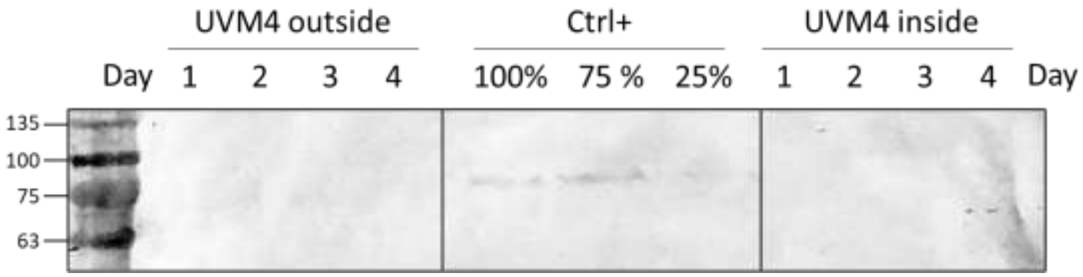

Supplementary Figure 6. Western blot on UVM4 cells and supernatant at different times of cultivation. Zeolin_YFP cells at day1 is used as technical positive control; percentage is referred to number of loaded cells.

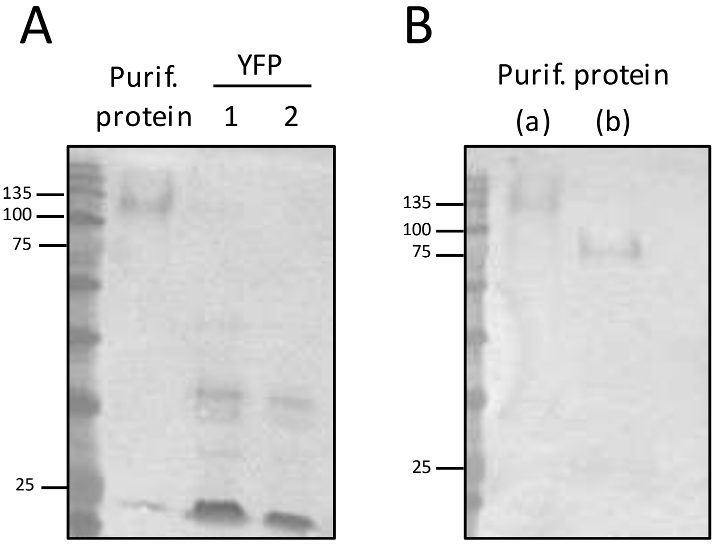

Supplementary Figure 7. Western blot analysis of zeolin-YFP complex. A) Recombinant zeolin-YFP was purified by affinity chromatography from exhausted growth obtained as supernatant upon centrifugation of cells expressing Bip_Zeolin:YFP vector cultivated in mixotrophy for four days. Purified YFP was used as standard (1=:0,005ng, 2=:0,0035ng) B) western blot on purified zeolin-YFP complex treated with 2M urea (a) or 2M urea and incubated for 5 minutes at 100°C (b)
